# Age-associated declining of the regeneration potential of skeletal stem/progenitor cells

**DOI:** 10.3389/fphys.2023.1087254

**Published:** 2023-02-02

**Authors:** Luigi Mancinelli, Giuseppe Intini

**Affiliations:** ^1^ Department of Periodontics and Preventive Dentistry, University of Pittsburgh School of Dental Medicine, Pittsburgh, PA, United States; ^2^ Center for Craniofacial Regeneration, University of Pittsburgh School of Dental Medicine, Pittsburgh, PA, United States; ^3^ Department of Medicine (Hematology/Oncology), University of Pittsburgh School of Medicine, Pittsburgh, PA, United States; ^4^ University of Pittsburgh UPMC Hillman Cancer Center, Pittsburgh, PA, United States; ^5^ McGowan Institute for Regenerative Medicine, University of Pittsburgh, Pittsburgh, PA, United States

**Keywords:** skeletal stem/progenitor cells (SSPCs), aging, bone regeneration, Prrx1/Prx1, senescence

## Abstract

Bone fractures represent a significant health burden worldwide, mainly because of the rising number of elderly people. As people become older, the risk and the frequency of bone fractures increase drastically. Such increase arises from loss of skeletal integrity and is also associated to a reduction of the bone regeneration potential. Central to loss of skeletal integrity and reduction of regeneration potential are the skeletal stem/progenitor cells (SSPCs), as they are responsible for the growth, regeneration, and repair of the bone tissue. However, the exact identity of the SSPCs has not yet been determined. Consequently, their functions, and especially dysfunctions, during aging have never been fully characterized. In this review, with the final goal of describing SSPCs dysfunctions associated to aging, we first discuss some of the most recent findings about their identification. Then, we focus on how SSPCs participate in the normal bone regeneration process and how aging can modify their regeneration potential, ultimately leading to age-associated bone fractures and lack of repair. Novel perspectives based on our experience are also provided.

## 1 Introduction

The skeleton is a complex apparatus made of several types of tissues, including bone, endothelium, cartilage, and adipose and hematopoietic tissues. Beyond its primary functions of structural support and movement, the skeleton houses the bone marrow, in which hematopoiesis occurs, and stores or releases minerals (Karsenty and Ferron, 2012). Skeleton, like all the other tissue systems in our body, as time goes by is subjected to a series of detrimental processes which slow down and reduce its physiological functions. Collectively, these processes are defined as “aging”. In modern society, the lifespan has increased progressively and, with that, the number of elderly people. As a result, the health burden of bone fractures and skeletal weaknesses has also raised significantly; consequently, scientific interest in skeletal health has increased worldwide. As mentioned, the skeleton is composed of many distinct cell types; yet, skeletal/stem progenitor cells (SSPCs) are fundamental to maintaining and regenerating the skeleton and therefore are the major subject of scientific interest.

To start studying and characterizing the SSPCs’ roles in bone homeostasis and diseases, first their identity should be completely unveiled. However, despite various efforts, so far there is no consensus about such identity (Ambrosi et al., 2019). Consequently, we still do not completely understand the basic cellular and molecular mechanisms underlying the bone regenerative potential and how such potential is affected by aging, leading to impaired healing. This review aims to explore the current knowledge about the aging of SSPCs and their loss of regeneration potential thorough aging. First, we will consider some of the most recent findings about SSPCs’ identity. We explore studies performed in both mice and humans, underling the many locations and sources of the SSPCs. These multiple studies suggest that perhaps more than one identity of SSPCs exists, underscoring an heterogeneity in terms of their anatomical location. Then, we focus on the mechanisms of aging that are responsible for the declining of the SSPCs regeneration potential, leading to the age-associated bone weakening, fractures, and impaired repair/regeneration. We describe the significance of employing new technologies, such as single-cell RNA sequencing (scRNA-seq), to better understand the biology of SSPCs, properly identifying them and comparing their functions and disfunctions. Finally, we introduce new perspectives, based on our experience in the field.

## 2 Identification of SSPCs: A fundamental issue

Stem cells present two fundamental characteristics: the ability to self-renew, which allows for their replenishment, and the capacity to differentiate into multiple cell types, which preside to tissue development and regeneration. Since Haeckel first used the term “stem cell” in the 19th century, these concepts have been largely accepted and experimentally verified, and the scientific community has made significant advancements in this field of research. For instance, stem cells have been identified in different tissues (hematopoietic (Ng and Alexander, 2017), neural (Takagi, 2016), epithelial (Visvader and Smith, 2011), etc.) and somatic cells can now be reprogrammed into pluripotent stem cells (Takahashi and Yamanaka, 2006).

SSPCs were first described simultaneously to the hematopoietic stem cells (HSCs), but their characterization has been much more difficult and controversial than the HSCs, perhaps because of their multiple anatomical locations. All began in the 60’ when a series of studies (Tavassoli and Crosby, 1968; Owen and Friedenstein, 1988) showed that bone marrow was able to regenerate bone, stroma and adipocytes, and support hematopoiesis. This ability was imputed to stem cells residing in the bone marrow. It took many years for the bone marrow derived stem cells to earn the name of SSPCs (for a complete revision about the origin of the skeletal stem/progenitor cells name, readers should refer to (Ambrosi et al., 2019)). Before consensus was achieved, SSPCs have been called first “Mesenchymal Stem Cells” (Caplan, 1991) and then “Mesenchymal Stromal Cells” (Dominici et al., 2006); the generic use of these names along with the various assays employed to prove their stem cells qualities, has contributed to generate confusion over the years (Ambrosi et al., 2019).

**FIGURE 1:**
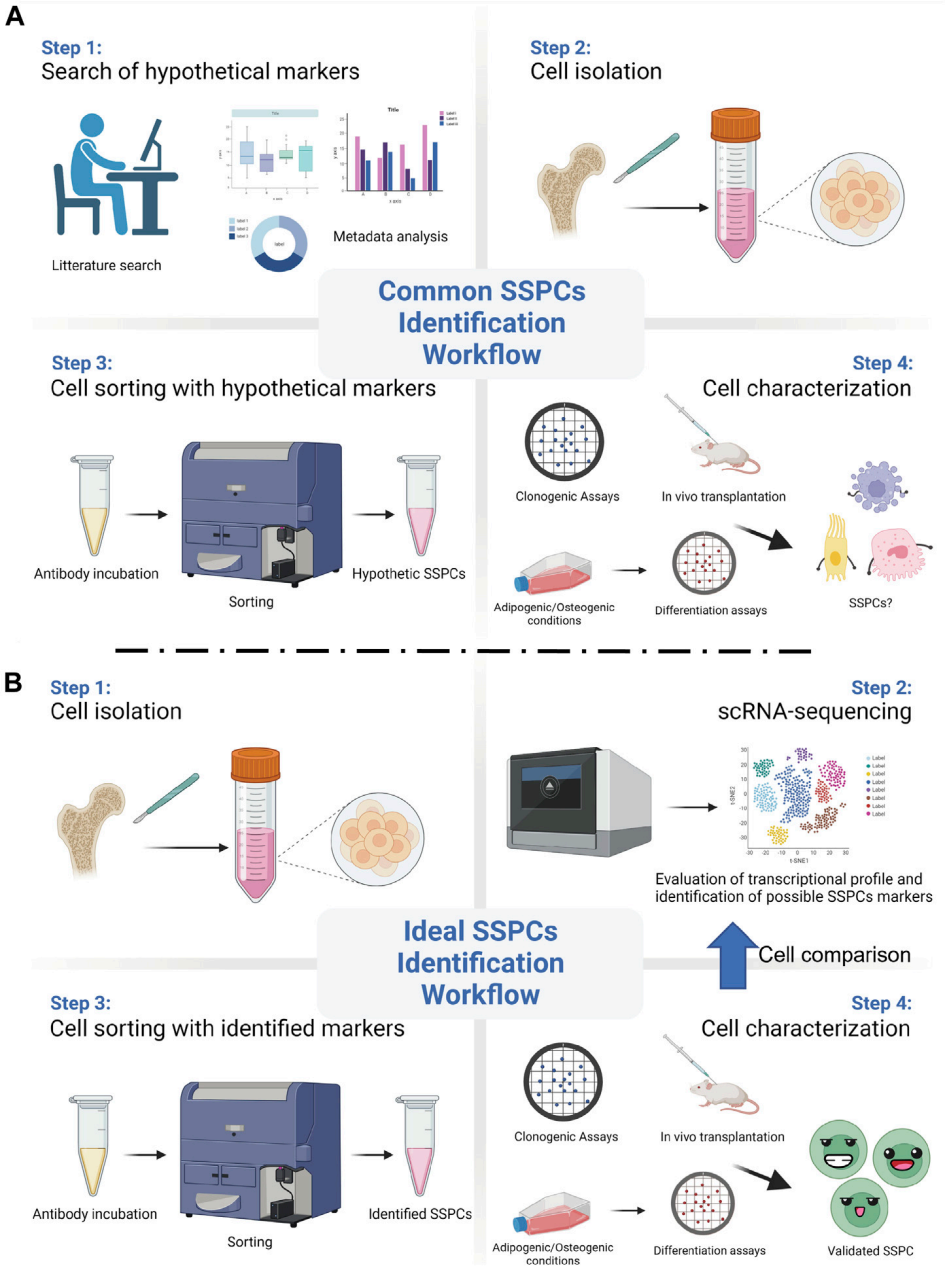
Common vs ideal SSPCs identification workflow. **(A)** A common and biased approach starts with the selection of SSPCs markers using previously published data. Then, such markers are used to sort hypothetic SSPCs from the pool of the isolated cells. Different assays are then employed to test the putative SSPCs self-renewal, clonogenic, and differentiation abilities. **(B)** Recent technological advancements, such as single-cell RNA sequencing (scRNA-seq), allows for an unbiased and ideal workflow that starts from the evaluation of the transcriptional profile and the identification of appropriate SSPCs markers right after cell isolation. After sorting them with the identified markers, SSPCs can be tested for their stem cell properties. (*Created with* *BioRender.com*).

The employment of powerful and reliable assays is crucial to identify stem cell properties in a putative SSPC population. Such assays should also be changed or updated as new technologies advance. For instance, today it is still common practice to utilize markers that were previously identified and that have not been validated with the new available technologies. With the advent of scRNA-seq, important information and details regarding the transcriptional profile of the analysed cells, which would reveal the expression of genes associated to their regeneration potential and their unique identity, can be unveiled. The ideal scRNA-seq workflow should begin with animal lineage tracing studies that identify putative stem cells. Then, an unbiased and reliable assessment of the transcriptional profile of the putative SSPCs should follow, with the final goal of identifying their surface markers. Once the surface markers have been discovered and validated, SSPCs can be reliably isolated, so that the evaluation and the characterization of their stem cells qualities, both *in vitro* and *in vivo*, can follow (Figure 1). The animal studies should be paralleled by human studies, so that the human homolog putative SSPCs can be isolated, identified, and characterized.

To date, various cell populations, from different regions of the skeleton, have been defined as SSPCs. As mentioned, first came cells isolated from the bone marrow cavity, which many refer to as “mesenchymal stem cells” (MSCs). This is the case, for instance, of Grem1+ cells and Lepr + cells, which have been previously reported to exhibit SSPCs qualities, such as the ability to form bone, cartilage, and adipose tissue (Zhou et al., 2014; Worthley et al., 2015). However, subsequent scRNA-seq analysis revealed lineage biases associated to the identification of these SSPCs (Baryawno et al., 2019; Tikhonova et al., 2019). For instance, expression of Lepr has been demonstrated to mark a wide and heterogeneous population of stromal cells, of which only a subgroup could be considered as authentic SSPCs (Baryawno et al., 2019). Even the expression of Cxcl12, which has been previously shown to largely overlap with the expression of Lepr (Ara et al., 2003), identifies a mixed population of cells that, when needed, for instance upon injury, converts into skeletal stem cell-like (Matsushita et al., 2020). Glioma-associated oncogene 1 (Gli1) is another marker that has been used to identify putative SSPCs of the bone marrow. However, Shy et al. found that Gli1+ cells, which co-express perilipin, a marker of adipocytes, and Lepr, are only present in high quantity in the marrow cavity of mice during embryogenesis (Shi et al., 2017). Postnatally, Gli1+ cells can be found mainly by the trabeculae and the growth plate. Only subsequently, by 9 months of age, Gli1+ cells reappear in the bone marrow, while decreasing by the trabeculae and the growth plate. This finding suggests that the postnatal Gli1+ cells of the bone marrow are stromal cells that derive from the postnatal Gli1+ SSPCs normally residing by the growth plate (Shi et al., 2017). Physiologically, bone marrow residing SSPCs have been described to possess a dual role: to constitute a reservoir of cells of the skeletal lineage for bone growth, and to support hematopoiesis (Bianco et al., 2013; Greenbaum et al., 2013). For instance, bone marrow residing SSPCs expressing CD146, described as adventitial reticular cells (ARCs), have been located by the sinusoids and have been shown to have this dual function (Sacchetti et al., 2007). However, these same studies also described cells that, while representing a reservoir of skeletal cells, are not able to support hematopoiesis (Sacchetti et al., 2007). This finding is also supported by subsequent studies showing that bone marrow residing SSPCs (labelled by the expression of Adiponectin) may have limited functions, as they might only be involved in the repair and regeneration of small and mechanically stable bone defect (Jeffery et al., 2022). A deeper analysis *via* scRNA-seq might be helpful to reveal the exact identity of the bone marrow SSPCs with dual function, to distinguish them from those only able to differentiate in cells of the skeletal lineage.

It is important to note that SSPCs found by the trabeculae and/or the endosteum are sometimes also labelled as SSPCs of the bone marrow. An example is represented by the SSPC population recently characterized by Liu et al. (Liu et al., 2022). These cells, identified by the expression of Paired related homeobox 1 (Prrx1), are defined as “bone marrow Prrx1+ SSPCs” even though they can be found by the trabeculae, the endosteum, and in the bone marrow. In this study the authors showed that genetic ablation of these “bone marrow Prrx1+ SSPCs” leads to an osteoporotic phenotype, reduction of trabecular bone number and bone volume, as well as to impaired bone healing (Liu et al., 2022). Importantly, the authors performed a scRNA-seq analysis of these cells, confirming that they express several markers commonly used to identify SSPCs. However, a scRNA-seq evaluation of the Prrx1 expressing cells isolated exclusively from the bone marrow could have revealed differences, or similarities, between these cells and the Prrx1 expressing cells of the endosteum and the trabeculae. This scRNA-seq approach would identify and validate markers of a specific population of SSPCs, thus providing the opportunity to characterize multiple types of SSPCs (Figure 1).

Being the location where cells in active proliferation mature into osteoblasts, the growth plate, which is responsible for the elongation of the long bones, has been proposed to be another location where SSPCs can be discovered. In fact, similar to what has been shown for the SSPCs of the bone marrow, different population of SSPCs can be found in the growth plate, and, as observed for the SSPCs of the bone marrow, the SSPCs of the growth plate also support bone formation and hematopoiesis (Chan et al., 2015; Mizuhashi et al., 2018). One of the most referred studies about SSPCs of the growth plate was performed by Chan and colleagues (Chan et al., 2015). Using a “Rainbow mouse” crossed with a mouse carrying a tamoxifen-inducible Cre recombinase under the control of the actin promoter, these authors revealed that within the mouse growth plate there is a clonal region of cells able to form bone, cartilage, and stromal tissue but not muscle, adipose, or hematopoietic tissue (Chan et al., 2015). Then, they isolated from the growth plates putative common progenitor cells by selecting for expression of hematopoietic (CD45 and Ter119), vascular (Tie2), and osteoblastic (Integrin alpha V/ItgaV) markers; they found that cells expressing ItgaV can be fractioned in eight sub-population of cells on the basis of the expression of CD105, Thy, 6C3, and CD200. After testing the ability of these sub-populations to self-renew and give rise to skeletal tissue, and after verifying whether any of these sub-populations was able to generate others, they concluded that CD45-Ter119-Tie2-ItgaV+Thy-6C3-CD105-CD200+ cells are the murine SSPCs of the growth plate. Taking a similar approach, the same authors identified human SSPCs (Chan et al., 2018). Such approach relied on the use of a pre-existing set of markers generated by a metadata analysis to validate the SSPCs’ traits of different population of cells. As mentioned above, in reference to the SSPCs identified in the bone marrow, once again an approach utilizing scRNA-seq to identify and validate markers of a putative population of SSPCs may provide the opportunity to widen the search for markers and perhaps identify multiple types of SSPCs (Figure 1). Another noteworthy investigation about SSPCs of the growth plate has been conducted by Mizuhashi et al. (Mizuhashi et al., 2018). This study utilized the panel of SSPCs markers proposed by Chan et al. (Chan et al., 2015) and characterized a unique class of SSPCs, originally unipotent and becoming multipotent at the post-mitotic stage. These cells are characterized by the expression of parathyroid hormone-related protein (PTHrP) and originate from a small subset of PTHrP + chondrocytes precisely located within the resting zone of the post-natal growth plate. The authors tested the self-renew and differentiation abilities of these cells, both *in vitro* and *in vivo*, and claimed their SSPC identity. A further fascinating aspect of these cells is that they are not found during fetal development, as they can be found only after the formation of the growth plate, suggesting that a distinct environment, which could be defined as a niche (see also Section 3.3 hereafter), is required for SSPCs development and self-renewal. Once again, with the final goal of validating a list of genes which could be used to sort and characterize these SSPCs for medical purposes, it would be extremely interesting to investigate through scRNA-seq the complete transcriptional profile of these growth plate SSPCs, characterizing their equalities or differences. Similar findings have been described by Newton and colleagues (Newton et al., 2019), who observed a shift in the clonality of chondrocytes of the growth plate. This shift is accompanied by a marked depletion of chondroprogenitors during the formation of the growth plate, and by the acquisition of self-renewal abilities as soon as the growth plate is formed. To better investigate this phenomenon, the authors used laser capture microdissection and single cell RNA Smart2 sequencing to compare chondroprogenitors isolated at P2 with chondroprogenitors isolated at P28. Interestingly, they found changes in genes related to the extracellular matrix, the oxidative stress, and the regulation of WNT and ERK1/2 pathways (Newton et al., 2019), indicating that gain of stemness is regulated by the niche microenvironment. Furthermore, the authors reported CD73 to be the most upregulated “stem cell surface marker” for these cells, and, consequently, performed experiments using CD73+/CD49e+ cells to study their potential to differentiate in chondrocytes, osteoblasts, and adipocytes (Newton et al., 2019). Leveraging more on the potentials of scRNA-seq analysis could have led the authors to the identifications of additional differences between the P2 and the P28 chondroprogenitor cells.

Another anatomical region that contains dividing cells and that, for this reason, has been of interest in SSPCs research is the periosteum. The periosteum is a thin fibrous membrane that lines the outer surface of bones and is made of fibroblasts, extracellular matrix, blood vessels, nerves, and, in the inner cambium layer, of osteoblasts and SSPCs (for a thorough review of biology and applications of the periosteum, readers may refer to (Lin et al., 2014)). The potential of the periosteum to generate bone after a fracture was firstly reported by Dr. Alexander Watson (Watson, 1845). Additional studies, where loss or damage of the periosteum was associated to lack of fracture repair further supported the initial observations of Dr. Watson (Garcia et al., 2008; Wu et al., 2021). Very recently, a study compared the contribution to the repair of various types of injuries between the bone marrow Adiponectin + SSPCs and the periosteal Gli1+ SSPCs (Jeffery et al., 2022). While, as mentioned above, the Adiponectin + SSPCs of the bone marrow are involved with the repair and regeneration of small and mechanically stable bone defects, the periosteal Gli1+ SSPCs are involved with the repair the bicortical fractures, suggesting that, depending on type of damage and mechanism of repair, distinctive SSPCs are required. Thus, different SSPCs have different abilities which may or may not be necessarily related with their potency. Identifying SSPCs in the periosteum is difficult because the periosteum is thin, with limited cellularity, and difficult to collect. In fact, current methods to extract cells from the periosteum are based on mechanical scraping and subsequent enzymatic digestion; alternatively, periosteal cells have been isolated by means of direct *ex-vivo* tissue culturing of the explanted periosteum (Roberts et al., 2015). To track periosteal SSPCs, studies have used countless markers (for a complete list, we suggest looking at (Perrin and Colnot, 2022)). Gli1 and Prrx1, which, as mentioned above, have been used to identify SSPCs of the bone marrow, along with Axis inhibition protein 2 (Axin2) and Cathepsin K (Ctsk) have also been utilized to mark SSPCs of the periosteum. In fact, expression of both Gli1 and Axin2 has been found to mark cells with skeletogenic potential in mouse embryo and post-natal tissues (Zhao et al., 2015; Maruyama et al., 2016; Ransom et al., 2016). Ctsk, encoding for the cysteine protease cathepsin K and traditionally used as marker of the bone-resorbing osteoclasts, has now been reported to identify an SSPC population of the mouse periosteum (Debnath et al., 2018). Expression of Prrx1, a transcription factor that is highly expressed during limb bud formation and craniofacial development, has been utilized to identify SSPCs in the mouse periosteum (Duchamp de Lageneste et al., 2018), as well as in the mouse periodontium (Bassir et al., 2019) and the mouse calvarial sutures (Wilk et al., 2017).

Calvarial sutures is the latest site in which SSPCs have been found. Indeed, calvarial sutures are synarthrosis composed by fibrous and connective tissue that act not only as connectors between the calvarial bones but also as reservoir of SSPCs (Wilk et al., 2017). For instance, it has been shown that during calvarial bone development putative SSPCs of the calvaria expressing Msx2 are destined to remain undifferentiated within the most central portion of the suture while only cells next to the advancing osteogenic fronts get incorporated into the growing bone (Lana-Elola et al., 2007). This indicates that the fate of SSPCs may depend on their position within the suture. Our group has also shown that putative mouse SSPCs expressing Prrx1 reside in the calvarial suture niche, respond to WNT signalling both *in vitro* and *in vivo* by differentiating into osteoblasts, are required for calvarial bone regeneration, and, upon heterotopic transplantation, are able to regenerate calvarial bone (Wilk et al., 2017). Others have shown that SSPCs of the calvaria may also express Gli1, Axin2, and Ctsk (Zhao et al., 2015; Maruyama et al., 2016; Debnath et al., 2018). Recently, we have been able to isolate SSPCs resident within the calvaria, and, using scRNA-seq analysis, we have delineated their transcriptional profile. Following a scRNA-seq approach, we compared the expression of all the known potential markers of SSPCs, including CD146, 6C3, CD200, and found that Ctsk, Gli1, Axin2, and Prrx1 are the only four genes whose expression is significantly overlapped in certain cells of the calvaria sutures (manuscript in revision). In this case, the scRNA-seq approach has allowed for a comprehensive analysis, permitting to evaluate the overlap of expression of the many previously proposed markers. Considering the large number of existing studies that describe different putative SSPCs, such comprehensive approach should be used to reach a consensus over the existence of a single or multiple populations of SSPCs within the same anatomical area. Moreover, since calvarial bones are formed *via* intramembraneous ossification and calvarial bone defect are repaired by a similar process, calvarial SSPCs may have to be listed as a class of their own, with a unique biological activity that may differ from that of other SSPCs. ScRNA-seq comparative studies along with functional assays may help understand the similarities and differences between the calvarial SSPCs and the SSPCs of other skeletal segments.

In summary, SSPCs can be found in many different anatomical regions. Since investigations reported that the skeletal system is incredibly plastic (Mizuhashi et al., 2018) and since the activity of different types of SSPCs depends on the necessities (i.e., physiological or regenerative), we suggest that indeed multiple kinds of SSPCs may exist within the same anatomical site or in separate sites and that they act according to the skeleton urgencies. Thus, some of them may contribute more on supporting regeneration rather than hematopoiesis or *vice versa*. Unfortunately, no studies analysing functional similarities and differences in SSPCs have been performed to date. Therefore, comparing all putative populations of SSPCs is, in our opinion, a necessary exercise to identify common features across various SSPCs, finally solving the problem of SSPCs’ identification and characterization. scRNA-seq technology, by offering the opportunity to do so, should be systematically utilized for such guided approach to unify the available data.

## 3 Mechanisms of SSPCs aging and their impact on bone repair

As mentioned above, no consensus exists yet about the identity of SSPCs. Consequently, any consideration about the effects of aging on these cells can either be generic or can only be specific about a certain population of the putative SSPCs so far identified. Here we attempt such analysis, on the basis on the available evidence.

Aging is associated with the degenerative processes that physiologically occur in an organism as time goes by. Degenerative processes become evident after the organism reaches the reproductive age, suggesting that after fulfilling the main purpose of life, which is the perpetuation of the species, an organism is somehow programmed to deteriorate. These processes involve all organs, and cells of the body; thus, bone is not spared. Aged bones present with a lower bone mass, alterations in the number and the architecture of trabeculae (Link et al., 2002; Boutroy et al., 2005; Sornay-Rendu et al., 2007), as well as increase in matrix mineralization, which make them stiffer, but more brittle (Currey, 1990; Grynpas, 1993).

**FIGURE 2:**
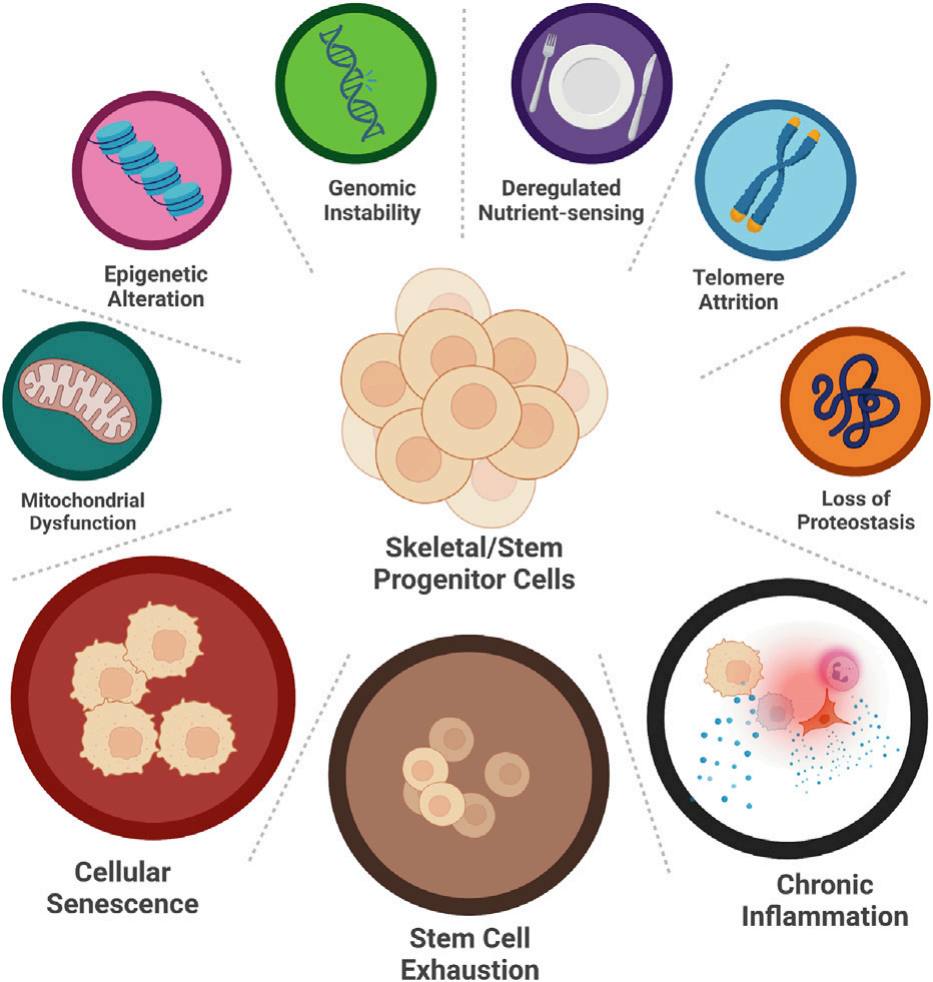
Hallmarks of SSPCs aging. Nine hallmarks of aging have been identified: genomic instability, deregulated nutrient-sensing, telomere attrition, loss of proteostasis, epigenetic alteration, mitochondrial dysfunction, cellular senescence, stem cell exhaustion and chronic inflammation. Only three of them, namely cellular senescence, stem cell exhaustion, and chronic inflammation, have been studied in aging SSPCs. (*Created with BioRender.com*).

Aging is a multifactorial process with many driving mechanisms involved; not all of them are fully elucidated and therefore aging, *per se*, is difficult to define. These mechanisms are called aging hallmarks, and, as per today, nine have been identified: genomic instability, deregulated nutrient-sensing, telomere attrition, epigenetic alterations, loss of proteostasis, mitochondrial dysfunction, inflammation, cellular senescence, and stem cell exhaustion (Figure 2) (Lopez-Otin et al., 2013). These hallmarks are highly interconnected: one can be both the cause and the consequence of another, and all together carry on the process of aging. For instance, inflammation can cause genomic instability, which can trigger cellular senescence, which in return can foster inflammation. Moreover, each of them is capable of directly and significantly influence aging, at least experimentally (Lopez-Otin et al., 2013), therefore it is very difficult to understand whether any of them has any dominant role, being responsible for triggering the others. In short, this is also the reason why aging is so difficult to intercept and why it is preferrable to attempt curing age-related diseases instead.

Unfortunately, studies about the aging hallmarks of the SSPCs are limited. Only few of them are available (Figure 2). Hereafter, we explore the results of these studies and report on their significance.

### 3.1 Stem cells exhaustion

**FIGURE 3:**
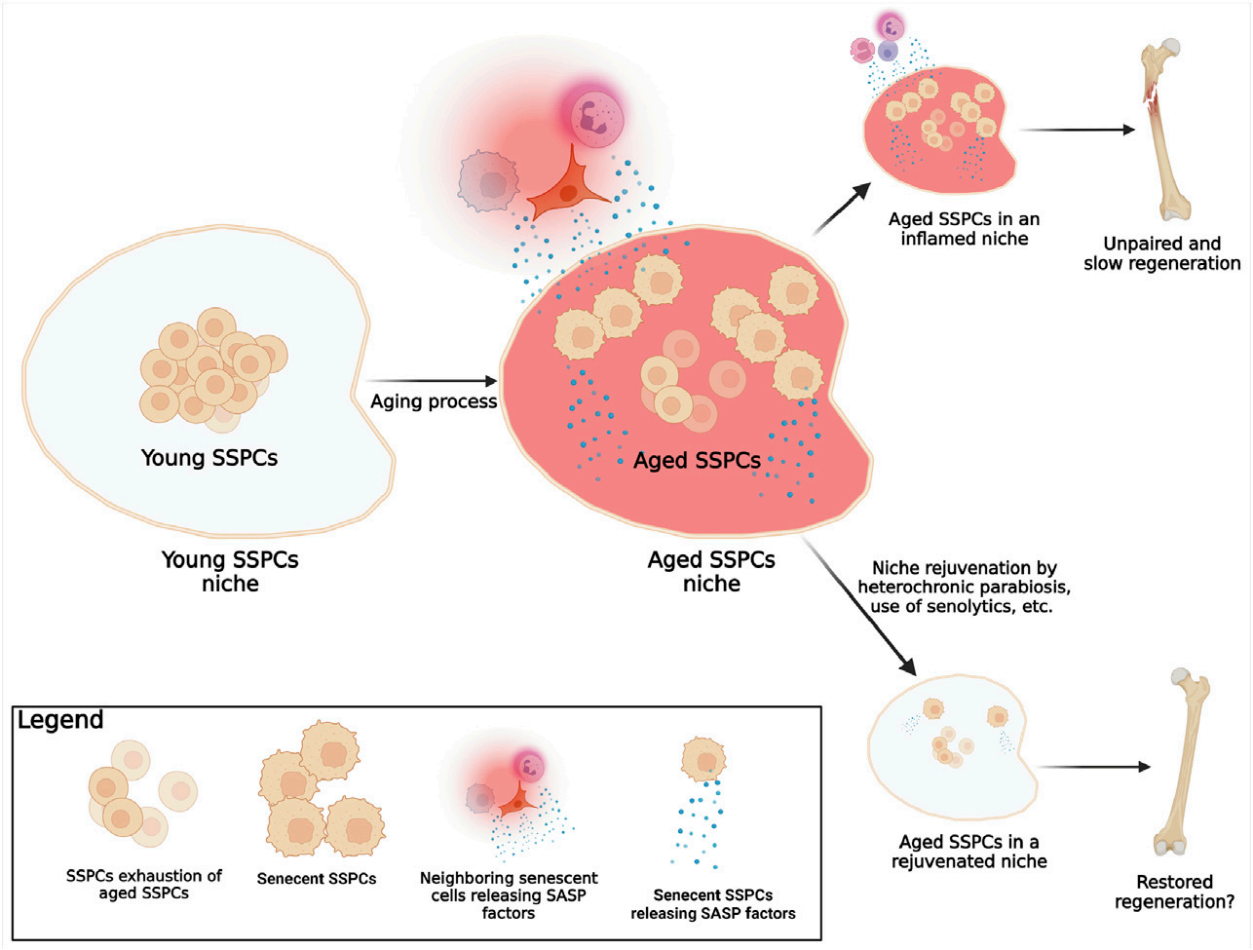
Known mechanism of SSPCs aging. In a young organism, the pool of young SSPCs is large, fully functional, and located in a young microenvironment (SSPCs niche) that offers ideal conditions for the SSPCs functions. As the organism becomes older, several mechanisms associated with aging and with a decline of SSPCs regeneration potential are known to happen: 1) the number of the now-aged, but probably still functional, SSPCs reduces (SSPCs exhaustion); 2) some SSPCs become senescent; 3) SSPCs niche conditions change (aged SSPCs niche) due to the chronic inflammation caused by the actively release of SASP factors by both senescent SSPCs within the niche, and neighboring senescent cells (immune, epithelial, endothelial, etc.). In the event of a fracture in elderly individuals the reduced number of SSPCs, the presence of senescent SSPCs, and the chronic inflammation within the aged niche are responsible for the lack of bone formation and fracture healing; a rejuvenation of the niche, by eliminating the inflammation and increasing the number of the functional non-senescent SSPCs, can foster fracture healing. (*Created with BioRender.com*).

The first aging hallmark constantly found active in the aging tissues where SSPCs reside (i.e., the bone marrow cavity, the growth plate, the periosteum, the calvarial sutures, etc.) is the stem cells exhaustion. This appears to involve all different populations of putative SSPCs identified so far (Martin and Olson, 2000; Farr et al., 2017) (Figure 3). The number of SSPCs available at the site of injury is crucial for proper bone healing and bone regeneration. For instance, our group has demonstrated that a significant reduction of the number of the SSPCs of the calvarial suture impairs calvarial bone regeneration (Wilk et al., 2017); conversely, recently generated data (manuscript in revision) shows that increasing the number of SSPCs *via* suture expansion fosters regeneration of calvarial critical size defects, otherwise unable to spontaneously regenerate. These observations suggest that: 1) a minimal number of SSPCs is required to sustain and promote bone healing; 2) in an aged environment, stem cell exhaustion may be one of the primary reasons for the impaired bone regeneration; 3) the proliferation of SSPCs that occurs in an aged organism after an injury does not reach a proliferative threshold able to sustain regeneration. This may be due to an initial very limited number of cells or to their intrinsic ability to proliferate effectively. Thus, SSPCs may be depleted during aging not only by a reduction of their number, but also by senescence, a mechanism that impairs their vital functions (Figure 3).

### 3.2 Stem cells senescence

In biology, senescent cells are defined as cells that despite the presence of space, nutrients, and growth factors, stop proliferating but do not die (Hayflick, 1965). Traditionally, telomere shortening has been used to identify senescence (Carlone et al., 2021). This is the reason why studies finding no changes in telomerase activity in SSPCs (Ambrosi et al., 2021), concluded that senescence was not a significant player in aging of SSPCs. However, SSPCs may present other mechanisms by which they become senescent, despite the lack of changes in telomere length and telomerase activity. Two other mechanisms should be considered: 1) telomeres are just DNA ends, and therefore they can acquire damages and mutations that may result in cellular senescence independently from their length or the expression of telomerase (Kruk et al., 1995). Moreover, telomeres are invisible to DNA repair machinery, so their probability to develop a biologically significant mutation is higher when compared to other DNA regions (Griffith et al., 1999); 2) non-telomeric DNA damage accumulation may occur with aging (Burkhalter et al., 2015) (genomic instability) or *via* senescence associated secretory phenotype (SASP) factors released by other nearby senescent cells (Josephson et al., 2019). Unfortunately, no investigation has thoroughly analysed the characteristics of senescent SSPCs, and of course, this is also due to the lack of consensus about their identity. Despite these gaps in the literature, and thanks to few available findings about aged SSPCs and on other types of stem cells (Boyle et al., 2007; Coppe et al., 2010; Sousa-Victor et al., 2014; Ambrosi et al., 2020), we can at least speculate that senescent SSPCs are characterized by cell cycle arrest, apoptosis resistance, alterations in the expression of senescence specific genes such as CDKN1A, CDKN2A (Sharpless and DePinho, 2007), SIRT1, etc., and by the active production and secretion of SASP factors (Josephson et al., 2019). These speculations will require additional investigations; yet, what remains biologically significant in aging, is the existence of a certain number of senescent SSPCs that may reach a biologically significant threshold. Indeed, a single or few senescent cells may not be biologically significant because they can be eliminated by immune cells; yet, when the number of senescent cells raises exponentially, a series of deleterious events occur: first, since senescent SSPCs do not proliferate (Hayflick, 1965), the regenerative capacity of the tissue is compromised; second, once SSPCs become senescent, they lose their original identity and function, affecting the homeostasis of the tissue; and third, if SSPCs released SASP factors, they also sustain the senescence of other SSPCs in a paracrine fashion, increasing local environment inflammation (Campisi and d'Adda di Fagagna, 2007) (Figure 3).

In conclusion, it is important to distinguish aged from senescent SSPCs. The first are cells present in an old organism that still possess their original identity and are therefore still able to perform their duties as stem cells; the second, originate in response to age-associated factors characteristic of an aged microenvironment, and are non-functional (Figure 3). In other words, when the skeleton becomes older, the number of SSPCs may decrease or their regenerative potential may decline and this may be due to a reduced number of SSPCs, and increased number of senescent SSPCs, or both. Similar with what has been shown with HSCs (Ho et al., 2017), re-creating the original SSPCs cellular microenvironment may induce an increase of their number or may re-establish their function.

### 3.3 Chronic inflammation: Bad environment makes bad SSPCs

Inflammation is a significant hallmark of aging (Ferrucci and Fabbri, 2018), and it is even more important in the field of aging of SSPCs and age-associated compromised bone healing. Indeed, the first response after a bone fracture is represented by an acute inflammation, which is essential for initiating fracture healing. It has been demonstrated that mice deficient in innate and adaptive immunity have substantially compromised endochondral bone repair (Rapp et al., 2016) and that inhibition of inflammation causes delays in fracture recovering (Gerstenfeld et al., 2003). Inflammation, at the site of injury, is useful not only because its chemotaxis on neutrophils and macrophages, which clean the site from debris, but also for mobilization of SSPCs, providing the topological information about the regenerative activity site. However, a distinguish needs to be made between acute or chronic inflammation. While an inflammation strong in intensity but lasting a relatively short amount of time (acute inflammation) is beneficial for all the reasons said above, a chronic, weak, but non-resolving inflammation is detrimental to fracture healing. There is much evidence that in conditions of chronic inflammation bone healing is impaired (Kayal et al., 2007). Amongst the plethora of conditions that are accompanied by chronic inflammation (i.e., diabetes, Alzheimer’s disease, cancer, etc.), there is aging, which probably is the most significant and most subtle among all, because sometimes it’s not even considered as a condition.

Low chronic inflammation always accompanies aging, and in fact the term “inflammaging” is commonly used to describe this association (Franceschi and Campisi, 2014). A significant amount of data show how inflammaging is capable of inducing a reduction in bone regeneration potential (Sebastian et al., 2005) and, conversely, how rejuvenation of the inflammatory system in aged animals can accelerate fracture repair (Lu et al., 2005; Xing et al., 2010). Josephson and colleagues firstly demonstrated a direct correlation between the number of SSPCs and the time of healing of a human bone fracture and they identified chronic local inflammation as the main factor responsible for the decline of the SSPCs number and function. As mentioned, while acute inflammation is necessary to engage SSPCs recruitment and support tissue repair, chronic inflammation needs to be reduced, so that homeostasis can be restored.

Recent investigations have supported the concept of inflammaging as the main driving force in SSPCs dysfunction during aging (Ambrosi et al., 2021). This idea is appealing because it also suggests that SASP factors (interleukins and cytokines), by which inflammation exerts its effects, may be able to directly influence stem cell fate. Thus, directly modulating SASP factors, or indirectly modulating their effects, may lead to novel and effective therapeutic strategies in the field of bone regeneration.

Inflammation can also indirectly affect SSPCs functionality by affecting the environment in which they are located. In fact, stem cells reside in a specialized microenvironment (the niche), which, by means of self-renewal-regulating signals, adhesion molecules, and other cell types, conditions the properties and spatial organization of the SSPCs, maintaining their biological health and tissue competency. In addition, the niche provides an isolated space in which stem cells are kept safe from mechanical stimulations and from other damaging agents such as ROS and radiations. Unfortunately, as discussed above, a niche is not able to shield SSPCs from age-associated inflammation. In fact, Song et al. (2012) . Observed that increased SASP factors in aged mice contribute to altering bone marrow niches, which are depleted of osteopontin (OPN), a factor known to preserve the polarity and the physiology of the SSPCs. Yet, not much is known about the effects of aging on the SSPCs niche. This missing information has become particularly significant since more than one SSPCs niche has been described (i.e., the endosteal/bone marrow niche, the periosteal niche, the calvarial suture niche). In fact, it is possible that aging influences the various niches in different and unique ways, highlighting the importance of studies that identify and characterize SSPCs and their niches.

Rejuvenating the niche may sound appealing as a method to increase the number and re-establish the function of SSPCs. However, it may not be an easy task to accomplish. For instance, several studies demonstrated that the exposure to a youthful circulation (i.e., by means of heterochronic parabiosis) can improve bone repair in older animals (Baht et al., 2015; Ma et al., 2022). However, recent findings utilizing the same approach showed no reversion of SSPCs aging and no improvement of bone mass or healing (Ambrosi et al., 2021). These controversial outcomes may be due to the distinct conditions tested (age of mice used, evaluation of bone mass with or without a fracture first, etc.) and not necessarily to lack of efficacy in the rejuvenation strategy. Therefore, the topic of niche rejuvenation, while appealing, needs additional extensive studies. An interesting idea would be based on a multi-intervention strategy: on one side, rejuvenate the niches (i.e., simulating the heterochronic parabiosis with transfusions of blood obtained from young individuals), on the other side reduce the number of senescent SSPCs by means of agents such as senolytics (Kirkland and Tchkonia, 2020) (Figure 3).

## 4 Conclusion and future perspectives

The latest years have been characterized by significant attempts to identify and describe SSPCs in multiple locations (Sacchetti et al., 2007; Chan et al., 2015; Wilk et al., 2017; Chan et al., 2018; Debnath et al., 2018; Mizuhashi et al., 2018; Newton et al., 2019; Matsushita et al., 2020). Despite these efforts, we still lack important information about their identity, or different identities, and about the interplay that they have with their niches, both in young individuals as well as during aging. Therefore, future efforts should aim to characterize and compare various putative SSPCs within the same niche and among all niches, with the final goal of generating a register of SSPCs helpful to study their biology and their regeneration potential. Then, each SSPCs type can be studied in relation to aging or other conditions, such as diabetes. Emerging techniques like the scRNA-seq can help clarifying similarities and dissimilarities among putative SSPCs, figuring out biological properties of SSPCs and their unique identity.

The study of aged SSPCs is even more challenging than the young ones, since the old ones are quite rare (Wilk et al., 2017; Ambrosi et al., 2019; Ambrosi et al., 2020). As discussed above, along with the development of aging mechanisms, the chances of SSPCs becoming senescent grow. Thus, in an old organism the number of physiologically competent SSPCs is limited, while the number of senescent ones is higher. From this point of view, given their higher number, senescent SSPCs may be easier to study; yet the difficulty of recognizing them, since they probably share some features of the non-senescent/functional SSPCs from which they derive, remains a crucial problem that needs to be overcome.

Watchful readers may have noticed that, even if we mentioned nine hallmarks of aging, we only discussed three of them. This does not mean that the other ones do not or may not have a significant role during SSPCs aging. Simply, it’s just that no exhaustive investigations exist on SSPCs and aging, and this is also probably due to our lack of knowledge about the various SSPCs identity. Therefore, studies conducted in the past years suffer from this limitation; however, they may be still useful in suggesting interesting mechanisms of actions during aging.

As mentioned, given their limited number, studying SSPCs is extremely challenging, and even more so in aged organisms. Since not much is known about SSPCs, no significant strategies able to harness them for bone regeneration exist. In fact, most of the current approaches for bone regenerative therapies focus on the transplantation of bone competent cells or the implantation of osteoconductive or osteoinductive biomaterials (Borrelli et al., 2020). These approaches, which are not exempt from health risks, may not be necessary if the biological regenerative potential of SSPCs is fully exploited, so that they can be harnessed for autotherapies even in the elderly. To reach this goal, the precise identity of the SSPCs needs to be defined.
